# The relation between angioarchitectural factors of developmental venous anomaly and concomitant sporadic cavernous malformation

**DOI:** 10.1186/s12883-016-0691-3

**Published:** 2016-09-22

**Authors:** Tengfei Yu, Xing Liu, Xiangjiang Lin, Chuanfeng Bai, Jizong Zhao, Junting Zhang, Liwei Zhang, Zhen Wu, Shuo Wang, Yuanli Zhao, Guolu Meng

**Affiliations:** Department of Neurosurgery, Beijing Tiantan Hospital,Capital Medical University, China National Clinical Research Center for Neurological Diseases, Center of Stroke, Beijing Institute for Brain Disorders, Beijing Key Laboratory of Translational Medicine for Cerebrovascular Disease, 6 Tiantan Xili, Chongwen District, Beijing, 100050 People’s Republic of China

**Keywords:** Developmental venous anomaly (DVA), Cavernous malformation (CM), Angioarchitectural factors, Contrast-enhanced magnetic resonance imaging

## Abstract

**Background:**

Past studies found that cerebral developmental venous anomaly (DVA) is often concurrent with cavernous malformation (CM). But the reason of the concurrency remains unknown. The purpose of this study was to confirm whether angioarchitectural factors relate to the concurrence and which angioarchitectural factors can induce the concurrency.

**Methods:**

DVA cases were selected from the records of the same 3.0 T MR. The DVA cases was divided into two group which are DVA group and DVA concurrent with CM group. 8 angioarchitectural factors of the DVAs were selected and measured. Statistical analysis was performed by the Pearson chi-square statistic,analysis of variance (ANOVA) and multi-factor logistic regression analysis.

**Results:**

Five hundred three DVA lesions were found and 76 CM lesions coexisting with DVA. In the single factor analysis, all the 8 angioarchitectural factors of DVA were related to the concurrency. In the multivariate analysis, 6 angioarchitectural factors. Result of multi-factor logistic regression analysis is Logit(P) = -4.858-0.932(Location) + 1.616(Direction) + 1.757(Torsion) + 0.237(Number) + 2.119(Stenosis rate of medullary vein)-0.015(Angle), goodness of fit is 90.1 %.

**Conclusions:**

The angioarchitectural factors of DVA are related to the concurrency of DVA and CM. 6 angioarchitectural factors may induce the concurrency.

## Background

There are four major types of vascular malformations of the central nervous system: developmental venous anomaly (DVA), cavernous malformation (CM), arteriovenous malformation(AVM), and capillary telangiectasia [1]. Previous studies indicate that DVA and CM are the two most common central nervous system diseases among vascular malformations, and are frequently found coexisting.

DVA makes up most of all cerebral vascular malformations (CVM) and Our previous studies found that concurrent CM is more likely to form when a DVA has three or more medullary veins that are visible simultaneously in at least one MRI section [2–4].

Developmental venous anomaly (DVA) (also referred to as venous angiomas or venous malformations) is usually discovered incidentally on enhanced CT or brain magnetic resonance imaging (MRI) with a reported incidence of 0.05–2.56 % in the general population [5, 6]. DVAs are a congenital abnormality of venous drainage [7]. The lesion are composed of radially arranged venous complexes converging to a centrally located venous trunk, which drains the normal brain parenchyma [8]. Characteristically, DVAs have numerous dilated deep medullary veins presenting in “spoke wheel” or caput medusae configurations, which drain into a few dilated deep and/or superficial veins [9].

CMs are discrete well-circumscribed lesions formed by sinusoidal vascular spaces lined by a thin, single layer of endothelium of varying size and separated by a collagenous matrix devoid of elastin, smooth muscle, or other vascular wall elements [4, 10]. They lack the microscopic features of arteries or veins.

CM is reported in the literature to have an association with DVA at a rate of 2–33 % [7, 11–14]. Although there are no acknowledge theory to explain the anomaly high rate. Some studies show that the angioarchitectural factors of DVA are related to the concurrency of DVA and CM [2, 15]. High vein pressure and flow disturbance within the territory of DVA by the anatomical angioarchitectural factors may be key factors in leading to a cascade of events and subsequent development of a CM lesion [15]. But there are only a few correlative researches, which just studied a little part of angioarchitectural factors. In this study, analyze the factors (especially the angioarchitectural factors of DVA) associated with concurrency of these two diseases was aimed.

## Methods

### Study population and data collection

From January 1, 2007 to December 31, 2012, data were collected from the patients’ MRI registration system and MRI reports form the same 3.0 T MRI unit at Beijing Tiantan Hospital. During the study period, the following MRI machines used were: GE Signa 3.0 T, superconducting magnetic resonance imager. The contrast agent was Gadopentetate dimeglumine (Gd—DTPA). At a rate of 1 ml/sec via an 18-gauge peripheral intravenous catheter, Gd—DTPA was bolus-injected by an MR power injector. The dose was 0.2 mmol/kg. After 8 s of delay from the start of injection, the images were acquired.

For patients who underwent multiple MRI screenings over the study period, only the most recent screening results were included in this analysis. According to the standard procedures of Beijing Tiantan Hospital, all MRI images were analysed by two radiologists. The final diagnosis was approved by both radiologists. In rare cases when the radiologists’ diagnosis was inconclusive, the researcher examined the original MRI images and assigned a classification to the case.

### Diagnostic criteria using MRI

DVA diagnostic criteria included presence of lesions in the white matter, typical stellate or linear vascular lesions converging into a collecting vein and draining into the dural, sinus, or deep veins, and an umbrella or caput medusa-like appearance especially on an enhanced image [7].

CM diagnostic criteria included presence of lesions with reticulated mixed signal blood-containing locules with the classic heterogeneous “popcorn” appearance on both T1 and T2-weighted images, a rim of haemosiderin in the surrounding brain parenchyma; and minimal or no enhancement on the T1 image [7, 9]. Their appearance on a MRI will depend on the degree of the hemorrhage, with T2-weighted images being the most sensitive sequence. On imaging, the diagnosis of CM is one of exclusion; other causes of a single haemorrhagic lesion, such as arteriovenous malformation, bland intraparenchymal haemorrhage, haemorrhagic infection, and neoplasm must be excluded [16].

**Fig. 1:**
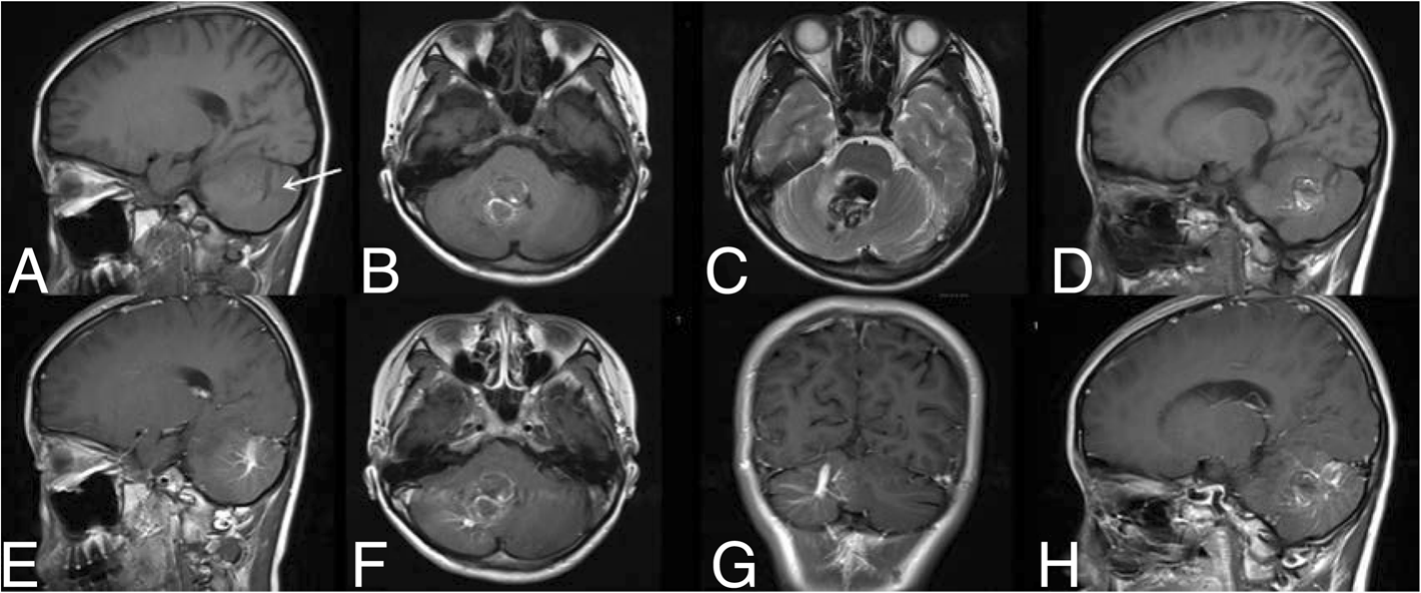
This is a 18-year-old female. It’s the typical MRI images for DVA coexist with CM. **a b c d** is the T1 weighted image, and **e f g h** is the enhanced image. The arrow refers to DVA

Hemorrhages were not classified as CMs if the hemorrhage lesions were only acute or subacute hematomas dominated by intracellular methaemoglobin, and therefore, appeared with a homogeneous signal on MRI images; and if there were only tiny, punctate foci of hypo intensity on both T1 and T2-weighted sequences, with no heterogeneous signal. On imaging, when CM diagnosis was made, other causes of a single hemorrhagic lesion, such as arteriovenous malformation, bland intraparenchymal hemorrhage, hemorrhagic infection, and neoplasm had to be excluded [17]. Figure 1 (This is a 18-year-old femal. It’s the typical MRI images for DVA coexist with CM.)

### Image analysis

All MR images should include:1 transverse and reformatted sagittal, coronal enhanced T1-weighted images .2 transverse and reformatted sagittal T1-weighted images. 3 transverse T2-weighted images. They were analyzed by two researchers retrospectively, using the Picture Archiving and Communication Systems (PACS) without any prior information. The final result was the mean value of two researchers’ measurement [17–20]. All the length, diameter, angle were measured by tools form the PACS.

Data of 8 angioarchitectural factors were collected. They are location of DVA(supratentorial or infratentorial), direction of the draining vein, torsion of draining vein, number of medullary veins, stenosis rate of draining vein, stenosis rate of medullary veins, length of draining vein, angle of the draining vein. Defination of the 8 angioarchitectural factors is as follow.

#### Factor 1 location of DVA

Two types of the location of the DVA lesion were defined, which were supratentorial or infratentorial.

**Fig. 2:**
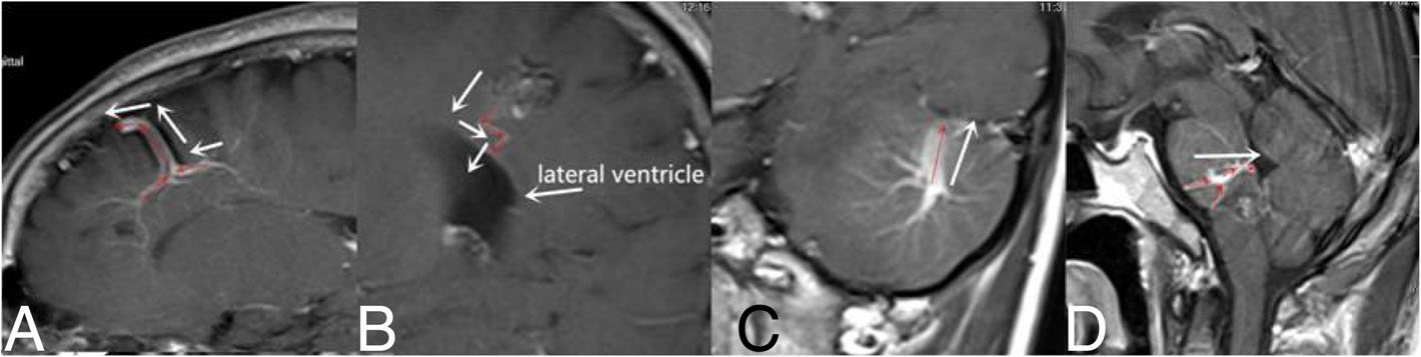
**a** is a 57-year-old DVA female patient. **b** is a 41-year-old DVA with concurrent CM male patient. **c** is the same patient with Fig. 1. **d** is a 35 years-old DVA male patient. The red arrows in **a**, **b**, **c**, **d** is the tool of PACS and it show the direction of draining. **a** is supratentorial DVA and superficial draining, **b** is supratentorial DVA and deep draining, **c** is infratentorial DVA and superficial draining, **d** is infratentorial DVA and deep draining

Factor 2 Direction of the draining vein Fig. 2 (A is a 57-year-old DVA female patient. B is a 41-year-old DVA with concurrent CM male patient. C is the same patient with Fig. 1. D is a 35 years-old DVA male patient. The red arrows in A,B,C,D is the tool of PACS and it show the direction of draining. A is supratentorial DVA and superficial draining, B is supratentorial DVA and deep draining, C is infratentorial DVA and superficial draining, D is infratentorial DVA and deep draining.)

The terminal or draining vein to which the caput medusae joins was classified as either a deep or superficial draining vein.

DVAs were defined as vascular lesions with multiple enlarged medullary veins converging on a single (sometimes multiple) dilated draining vein. All the DVA lesions have just one single draining vein in this research. So all six MRI sequences were analyzed and classified them. In the supratentorial compartment, superficial draining veins were identified as those that joined either a cortical vein or the sagittal sinus. Deep draining veins were identified as those that joined the subependymal veins of the lateral ventricles and ultimately the vein of Galen.

In the infratentorial compartment, superficial draining veins were identified as those that joined the cerebellar hemispheric veins, superior and inferior vermian veins,transverse or sigmoid sinus, and torcula. Deep draining veins were those that joined the subependymal veins of the fourth ventricle and thus either the anterior or lateral transpontine veins, or laterally and inferiorly to the veins of the lateral recess of the fourth ventricle, or superiorly to the precentral cerebellar vein [3, 12].

**Fig. 3:**
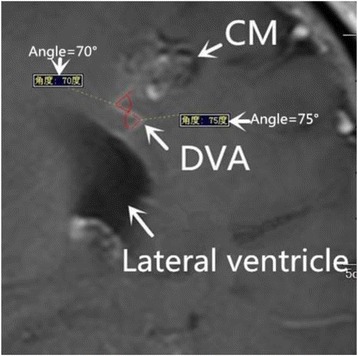
This is a 41-year-old patient who had DVA coexisting with CM. In this enhanced T1 image, two angles were found. Using the PACS tool(the red angles and yellow numbers), their angle were measured which are 70°and 75°. This DVA lesion had two angle less than 120°in the same section so it was defined as having torsion of the draining vein. Also the angle of draining vein was measured as 70°

Factor 3 Torsion of draining vein Fig. 3 (This is a 41-year-old patient who had DVA coexisting with CM. In this enhanced T1 image, two angles were found. Using the PACS tool(the red angles and yellow numbers), their angle were measured which are 70°and 75°. This DVA lesion had two angle less than 120°in the same section so it was defined as having torsion of the draining vein. Also the angle of draining vein was measured as 70°.)

Clinical routine MRI sequences was used for measuring the torsion. Because the are much more easily available than the 3D vision of draining vein. So the torsion factor was defined as this.

If the draining vein of DVAs has at least two angles less than 120°in the same one section of the enhanced T1 MRI sequences, it was considered to have draining venous tortuosity.

**Fig. 4:**
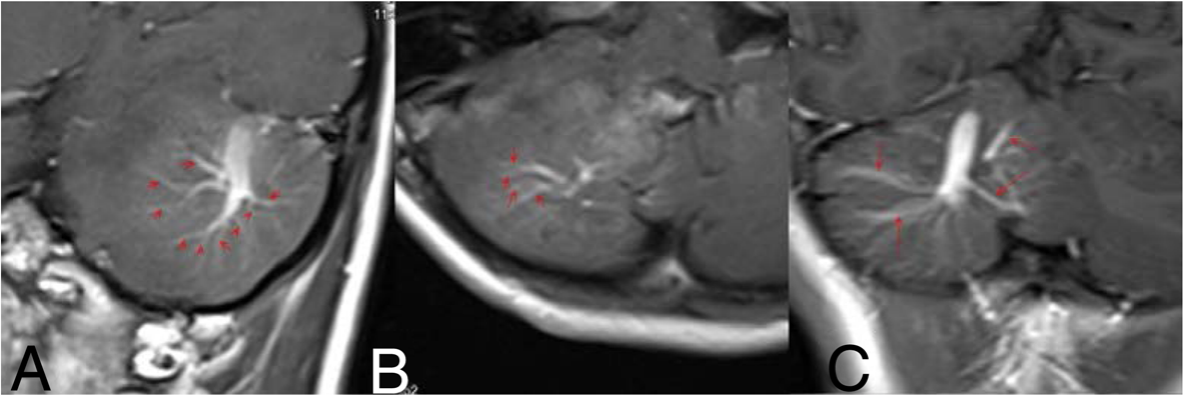
This is the same patient with Fig. 1. The red arrows show the medullary veins of the DVAs. **a** (9), **b** (4), **c** (4) had the most medullary veins in their sequences respectively. **a** had the most ones. So the number of medullary veins of this patient is 9

Factor 4 Number of medullary veins Fig. 4 (This is the same patient with Fig. 1. The red arrows show the medullary veins of the DVAs. A(9)、B(4)、C(4) had the most medullary veins in their sequences respectively. A had the most ones. So the number of medullary veins of this patient is 9).

A typical DVA lesion contains multiple enlarged medullary veins and a single (sometimes multiple) dilated draining vein. So number of the medullary veins of DVAs was defined as one of the 8 angioarchitectural factors. From different sections of enhanced T1 images, the DVA has different numbers of medullary veins. The maximum value of the numbers found from all the enhanced T1 images was defined as the number of the medullary veins of the DVAs.

**Fig. 5:**
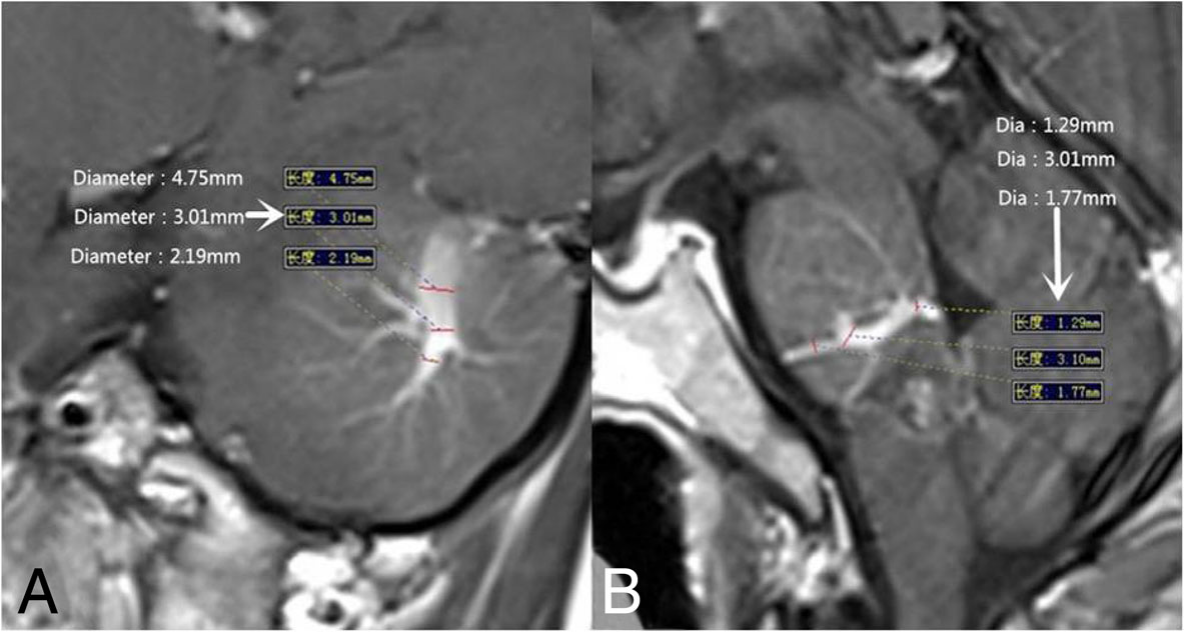
**a** is the same patient with Fig. 1. **b** is a 35-year-old patient who had DVA coexisting with CM. Both of them are enhanced T1 images. In **b** the wildest diameter of medullary veins is 1.77 mm, the wildest diameter of draining vein is 3.10 mm, the narrowest diameter of draining vein is 1.29 mm. So in **b** the stenosis rate of draining vein is (1-1.29/3.10) × 100 % = 58.39 %, the stenosis rate of medullary vein is (1-1.77/3.10) × 100 % = 42.90 %. In A, the same way was used

Factor 5 Stenosis rate of draining vein Fig. 5 (A is the same patient with Fig. 1. B is a 35-year-old patient who had DVA coexisting with CM. Both of them are enhanced T1 images. In B the wildest diameter of medullary veins is 1.77 mm, the wildest diameter of draining vein is 3.10 mm, the narrowest diameter of draining vein is 1.29 mm. So in B the stenosis rate of draining vein is (1-1.29/3.10) × 100 % = 58.39 %, the stenosis rate of medullary vein is (1-1.77/3.10) × 100 % = 42.90 %. In A, the same way was used).

In a DVA lesion, multiple enlarged medullary veins converging on a single dilated draining vein, which joins a sinus. According to the literature we put forward the hypothesis that stenosis rate of the draining vein can lead to the raise of venous pressure of DVA [11].

Using the PACS the widest and narrowest diameter of draining vein of all sections of enhanced T1 images were measured. The two diameters can be in the same section or different sections. Stenosis rate of the draining vein = (1- narrowest diameter/widest diameter) × 100 %.

#### Factor 6 Stenosis rate of medullary vein Fig. 5

Similar to the Factor 5, the widest diameter of draining vein and medullary veins of all sections of enhanced T1 images were measured. The two diameters can be in the same section or different sections. Stenosis rate of the medullary veins = (1- widest diameter of medullary veins/ widest diameter of draining vein) × 100 %.

#### Factor 7 length of draining vein

The caput of DVA is the part where medullary veins join the draining vein. The length of the caput to the end of draining vein is recorded in every section of enhanced T1 images. For the same reason of Factor 3 the maximum value was defined as the length of draining vein. The length of draining vein may have influence on the venous pressure [2, 3, 15]. If the draining vein was not straight, the length was measured part by part, using the PACS tools.

#### Factor 8 angle of the draining vein Fig. 3

Every angle of draining vein in all sections of enhanced T1 images were measured and define the minimum value as the angle of the draining vein.

### Statistical analyses

For single-factor analysis, statistical analysis was performed using the Pearson chi-square statistic for binary variables which are Factor 1,2,3 and analysis of variance (ANOVA) for continuous variable which are Factor 4, 5, 6, 7, 8.

Multi-factor logistic regression analysis were performed for predictors associated with the concurrency. In the Multi-factor logistic regression analysis, method was took as Forward:Conditional, Entry: *p* < 0.05, Removal: *p* > 0.10. 8 factors and independent variables included age, gender were analysised. For the age variable, age groups were formed by 20 year intervals (≤20, 20 ~ 40, 40 ~ 60, ≥60). All confidence intervals reported were 95 %, and all p-values were two-sided. P-values less than 0.05 were considered statistically significant. All statistical analyses were performed using SPSS software (Version 17.0). The dummy variables were Gender, Age, Location of DVA, Direction of draining vein, Torsion of draining vein.

## Results

Five hundred three DVA lesions met DVA radiographic criteria. 76 of 503 DVAs had coexisting CM and the other 427 DVAs lesion didn’t have coexisting CM. In the single factor analysis, all the 8 angioarchitectural factors of DVA were found related to the concurrency (*p* < 0.05) and age (*p* = 0.800) or gender (*p* = 0.627) had no relation with it. Compared with the DVA group,DVA concurrent with CM group were more likely to locate in infratentorial area,vein drain into the deep area, have torsion of draining vein and they tend to have more medullary veins, higher stenosis rate of draining vein and medullary veins, longer draining vein and smaller angle of draining vein Table 1.

**Table 1:** The result of single-factor analysis

		DVA with CM	DVA	P
Gender	Male	37 (48.7 %)	195 (45.7 %)	0.627
	Famle	39 (51.3 %)	232 (54.3 %)	
Age	<20	8 (10.5 %)	50 (11.7 %)	0.800
	20 ~ 40	29 (38.2 %)	180 (42.2 %)	
	40 ~ 60	34 (44.7 %)	165 (38.6 %)	
	≥60	5 (6.6 %)	32 (7.5 %)	
Location of DVA	supratentorial	35 (46.1 %)	327 (76.6 %)	<0.001
	infratentorial	41 (53.9 %)	100 (23.4 %)	
Direction of draining vein	Deep	57 (79.2 %)	181 (42.4 %)	<0.001
	Superficial	19 (20.8 %)	246 (57.6 %)	
Torsion of draining vein	Positive	50 (65.8 %)	42 (9.8 %)	<0.001
	Negative	26 (34.2 %)	385 (91.2 %)	
Number of medullary veins		5.91 ± 0.33	3.52 ± 0.08	<0.001
Stenosis rate of draining vein		53.66 ± 1.87 %	47.79 ± 0.68 %	<0.001
Stenosis rate of medullary veins		54.68 ± 1.85 %	43.65 ± 0.87 %	<0.001
Length of draining vein(mm)		21.45 ± 0.95	16.36 ± 0.40	<0.001
Angle of draining vein(°)		106.50 ± 3.84	136.62 ± 3.84	<0.001

In the multivariate analysis, 6 angioarchitectural factors including DVA location (supratentorial or infratentorial), the direction of the draining vein, whether there was torsion of the draining vein,number of the medullary veins, stenosis rate of the medullary veins, angle of the draining vein were associated with concurrency of DVA and CM.

Logit(P) = -4.858-0.932  (Location) + 1.616  (Direction) + 1.757  (Torsion) + 0.237  (Number) + 2.119  (Stenosis rate of medullary vein)-0.015  (Angle), goodness of fit is 90.1 %. Logit(P) is the result of Multi-factor logistic regression analysis and it implies the odds or risk of a DVA lesion being concurrent with CM. With the Logit(P) angioarchitectural factors can be transformed into numbers, which are more easily to tell a high risk DVA Table 2.

**Table 2:** The result of multi-factor logistic regression analysis

	B	S.E.	Wald	P	OR	95.0 % C.I.for OR	
						Lower	Upper
Location of DVA	−0.932	0.335	7.723	0.005	0.394	0.204	0.760
Number of medullary veins	0.237	0.078	9.165	0.002	1.267	1.087	1.477
Stenosis rate of medullary veins	2.119	1.076	3.876	0.049	8.321	1.009	68.591
Angle of draining vein	−0.015	0.006	5.310	0.021	0.985	0.973	0.998
Direction of draining vein	1.616	0.362	19.984	0.000	5.034	2.478	10.224
Torsion of draining vein	1.757	0.412	18.215	0.000	5.793	2.586	12.981
Constant	−4.858	1.125	18.643	0.000	0.008		

The angioarchitectural factors of DVA are related to the concurrency of DVA and CM. Especially, if the DVA follow the six angioarchitectural factors: locate in the infratentorial area, direction of the draining vein is deep, have torsion of draining vein, number of the medullary veins of DVAs ≥ 5, stenosis rate of the medullary veins ≥ 54.68 %, angle of the draining vein ≤ 106.50°,it tend to have concurrent CM.

## Discussion

Characteristically, DVAs have numerous dilated deep medullary veins presenting in “spoke wheel” or caput medusae configurations, which drain into a few dilated deep and/or superficial veins [21–23] MRI is an optimal imaging tool for detecting CM and DVA [15, 24]. So in this study the two lesion were connect with clinical routine enhanced MRI data, which is easily available in the hospital instead of some other MRI sequences.

In a significant percentage of DVA cases, however, coexisting vascular malformations are found. The most common vascular anomaly associated with a DVA is CM. The association between venous malformation and cavernous angioma has been recognized by many authors. In our previous study of 1839 patients, the rate is 11.15 % and 205 patients of the total 165,230 (1.24‰) was found had DVA with concurrent CM [2]. Reports suggested that DVA in the posterior fossa are more likely to hemorrhage than are their supratentorial counterparts [25]. Concurrent CM is more likely when a DVA is infratentorial. Contrast-enhanced MR images have clearly shown that cavernous angiomas frequently appear to arise at the distal radicles of venous malformations [2, 11, 14, 26].

Reasons for the concurrency may be related with the vein pressure of DVA. Cortes [4] agree with the theory that high venous pressure of DVA induce the form of CM. There is increased systemic or local venous pressure in the DVA [27]. Increased venous pressure may lead to recurrent petechial congestional hemorrhage [28, 29], or may produce ischemia which stimulates the growth of new vessels [30]. There is high venous pressure in the DVAs and high venous pressure and may be the cause of hemorrhage from a DVA with subsequent formation of a CM. Progressive thickening of the walls of the DVA and their morphological organization into a venous convergence zone are thought to contribute to the development of venous hypertension in DVA. These new vessels are fragile and susceptible to bleeding, and repeated hemorrhaging may subsequently form a CM [15, 28, 31]. Maeder P et al. [27] found a high percentage of venous stenosis of the collector vein in the patients who have CM in association with DVA. San MRD et al. [12] reported that outflow obstruction by stenosis of collecting vein was demonstrated in 13.1 % of the DVAs. Hong YJ et al [19]. found that anatomical angioarchitectural factors might be the key factors in causing concurrent sporadic CM within the territory of DVA by causing disturbance of blood flow. These factors include angulation of the vein, narrowing of distal draining vein, severe medullary venous tortuosity. Increased systemic venous pressure or increased local venous pressure secondary to stenosis of the draining transparenchymal vein or other venous obstruction could lead to haemorragic or ischaemic complications. Also lead to recurrent petechial hemorrhage characteristic of cavernous angiomas. Thrombosis of the collecting veins and raised pressure in the territory drained by the DVA are the causes of congestional haemorrhage. This, in turn, further provides angiogenic factor and stimulates growth of new vessels. Neovasculature lacks vasoregulatory capacity and is fragile enough to make it susceptible to bleed. This leads to repeated hemorrhage and hence formation of abnormal vessels and eventually a CM would be formed. Elevated venous pressure within the territory of a venous malformation may provoke angiogenic factors that may be responsible for the recruitment of new vessels. It is difficult to measure the pressure of DVA, but the pressure has been recorded in CMs during surgery and found to be substantially higher than cortical venous pressure.

So factors that increase the venous pressure can induce the form of CM. Dillon et al [32] reported that the development of cryptic vascular malformation was related to elevated venous pressure, occurring within a venous malformation, a telangiectasis, or a minute vascular malformation consequent to venous outflow obstruction. They found capillary telangiectasias and transitional lesions at the periphery of cavernous malformations in autopsy series and elevated venous pressure in DVAs leads to ectasia in an acquired telangiectasia that evolves toward a cavernous malformation. With this theory, we believe that higher risk of concurrency is related to higher risk of hemorrhage of CM.

Other reason for the concurrency may be: DVAs undergo changes common to the ageing venous system that can lead to ischaemic phenomena and secondary haemorrhage may then occur [28]. It is likely that cavernous malformations, as identified on MR imaging, are an end result of several possible initiating causes of vascular change: DVA, perhaps with stenosis in 1 branch; radiation; and genetic factors such as the KRIT1 and other known mutations [10].

DVA location (supratentorial or infratentorial), the direction of vein draining, torsion of draining vein, number of medullary veins, rate of stenosis of medullary veins, xangle of draining vein are the six angioarchitectural factors were found. Some studies show that the infratentorial DVAs may have higher venous pressure, because they are deep and have narrow space. When the draining vein have severe venous tortuosity, the venous pressure may be higher than other DVAs. With more medullary veins, DVA may have more venous blood in the lesion and that will lead to higher venous pressure. They can raise the venous pressure and thus raise the mobidity of DVA concurrent with sporadic CM. So angioarchitectural factors which cause disturbance of blood flow might be the key factors in causing concurrent sporadic CM within the territory of DVA [18, 33–35].

Here are the deficiencies of this research. The study focus on the MRI data of DVA and CM and didn’t contain the patients’ clinical symptom and sign or conditions of prognosis. Clinical correlations and detailed follow-up are limited and are beyond the scope of this imaging-based study. Because only the routine MRI sequences were used, the result data may have deviation with the true data of every angioarchitectural factors.

## Conclusions

This study shows that if the DVA follow the six angioarchitectural factors :locate in the infratentorial area, direction of the draining vein is deep, have torsion of draining vein, number of the medullary veins of DVAs ≥ 5, stenosis rate of the medullary veins ≥ 54.68 %, angle of the draining vein ≤ 106.50°,it tend to have concurrent sporadic CM. The concurrent morbidity can be predicted with the following equation. Logit(P) = -4.858-0.932  (Location) + 1.616  (Direction) + 1.757  (Torsion) + 0.237  (Number) + 2.119  (Stenosis rate of medullary vein)-0.015(Angle), goodness of fit is 90.1 %.

## Abbreviations

ANOVA: Analysis of variance
AVM: Arteriovenous malformation
CM: Cavernous malformation
CVM: Cerebral vascular malformations
DVA: Developmental venous anomaly
Gd—DTPA: Gadopentetate dimeglumine
MRI: Magnetic resonance imaging
PACS: Picture Archiving and Communication Systems
